# Supplementary material for: The phonon-modulated Jahn–Teller distortion of the nitrogen vacancy center in diamond
Source: Nat Commun. 2024 Oct 5;15:8646. doi: 10.1038/s41467-024-52712-9 (PMC11455962; doi:10.1038/s41467-024-52712-9)
Supplement: Supplementary file 2 — Supplementary Information [file 41467_2024_52712_MOESM2_ESM.pdf]

SUPPLEMENTARY INFORMATION

The Phonon-Modulated Jahn–Teller Distortion of the Nitrogen Vacancy Center in Diamond

*William P. Carbery, Camille A. Farfan, Ronald Ulbricht, and Daniel B. Turner**

Department of Chemistry, New York University, New York, New York 10003

Section Page Number

Supplementary Methods 4

Supplementary Discussion 9

Supplementary References.. 19

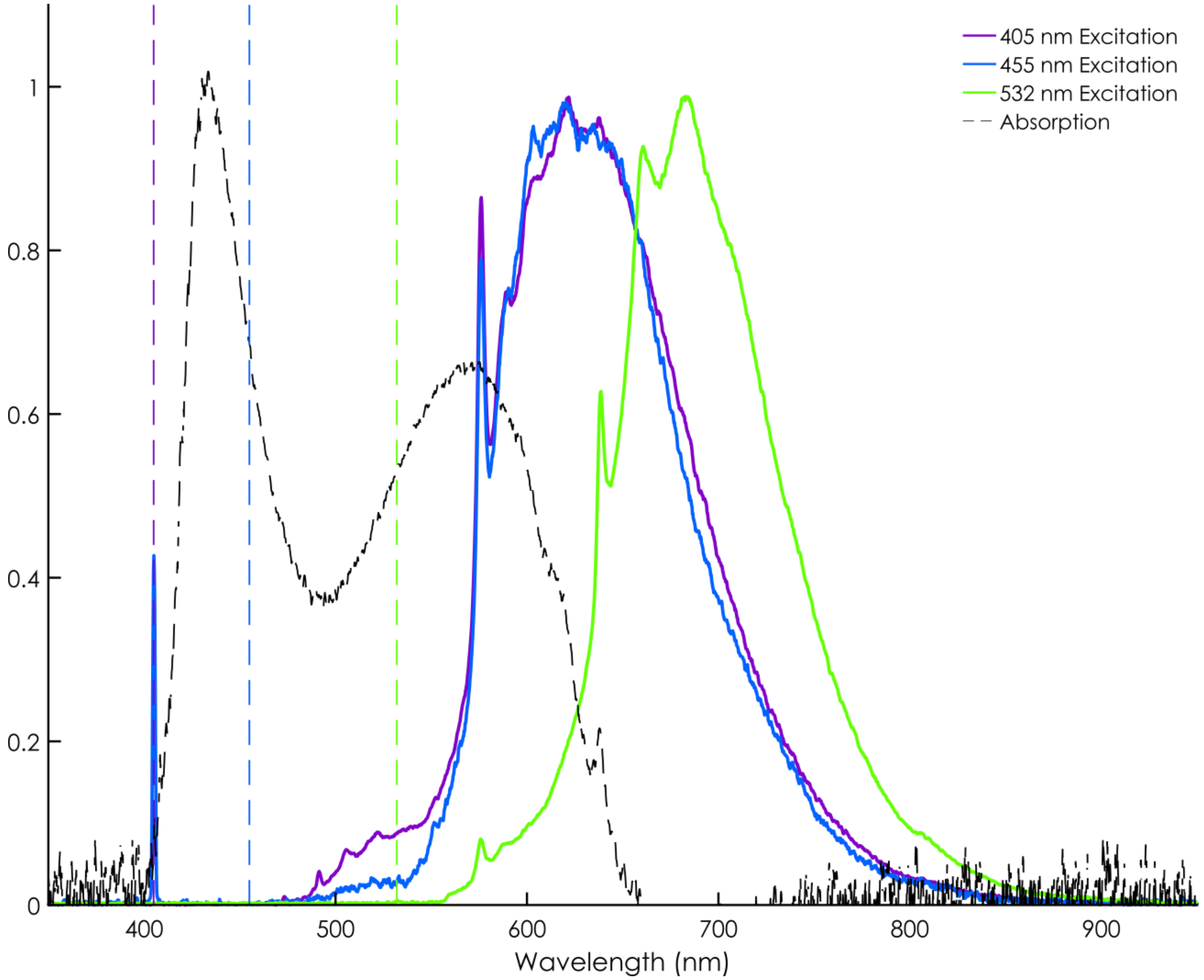

Supplementary Figure 1. Photoluminescence data on the NV sample used in the TA and 2D ES measurements.

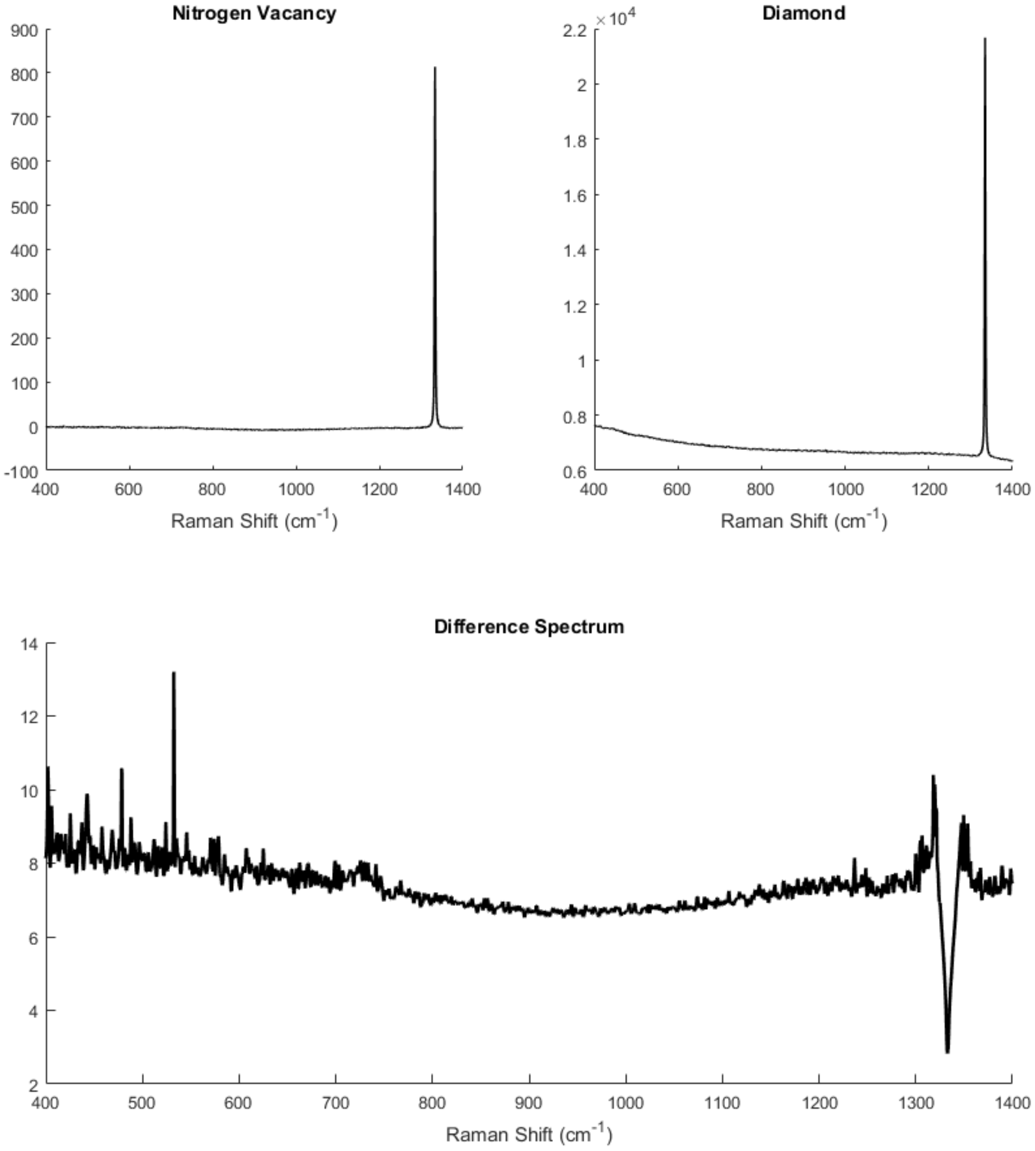

Supplementary Figure 2. Raman spectra of the NV center (top left) and a diamond blank (top right) used in the measurements for this report. The difference spectrum (bottom) shows nearly no variation between the two samples, except for a small, <1% signal below 600 cm^{-1}. The LO phonon at 1333 cm^{-1} (40 THz) is the predominant feature in both samples. Data taken using a Thermo-DXR2 Raman Microscope at 785 nm.

Supplementary Methods

The ultrafast spectroscopy setup used to measure the NV center in diamond is described in detail elsewhere [2, 3]. Briefly, a Ti:sapphire oscillator (80 MHz, 790 nm, 30 nm FWHM, 8 nJ) seeds a Ti:sapphire regenerative amplifier to yield laser pulses at a 1.25 kHz repetition rate (11 nm FWHM, 700 μJ, ~0.1% shot-to-shot relative standard deviation RSD). The amplifier output pumps a custom built noncollinear optical parametric amplifier (NOPA) that results in broadband laser pulses spanning 500-800 nm. For the NV center measurements, we modified our existing NOPA with an additional amplification stage tuned to the same spectral bandwidth. This resulted in pump and probe pulses of ~88 nJ with ~0.35% shot-to-shot RSD. Secondary amplification was necessary to achieve appreciable signal-to-noise ratios in the 2D ES measurements for the weakly absorbing NV center.

After amplification in the two-stage NOPA, the pulse is compressed using a prism-based pulse shaper to 6.5 fs as measured by transient-grating frequency resolved optical gating (TG-FROG). The setup uses two sets of chirped mirrors to over-chirp the higher-energy portion of the spectrum prior to interacting with a 39-channel linear membrane deformable mirror from OKO Tech. The deformable mirror is programmed with an optimization routine based on a machine learning algorithm trained on over 20,000 TG-FROG spectra obtained across years of recorded data using our specific NOPA and interferometer geometry [4]. For TA and 2D ES measurements, the compressed laser pulse is split into four beams in the forward BOX geometry. The probe/LO pulse is attenuated with a neutral density filter to be 10^4 less intense than the three pump beams. In the TA measurements used for `phasing' of the 2D spectra, the maximum nonlinear signal $\Delta T/T$ was 3%, and averaged over 2500 kinetic cycle pairs. We scanned the pump-probe interval from -0.5 to 12 ps in 1 fs steps. The acquired signal was free of nonresonant response after about 50 fs.

The above description of the NOPA and compression routine corresponds to the final state of the laser setup for the NV measurements most often presented in the main text. Some of the

datasets, specifically the 3D ES dataset taken with 1 fs steps, were collected before the machine-learning pulse compressor and dual-stage NOPA were implemented. As mentioned in the main text, the NV center presented unique challenges for ultrafast spectroscopy that took a three-year effort of instrument advancement to fully untangle. It was not until the pump and probe pulses became sub-8 fs with appreciable peak power that the vibronic peaks and LO phonon overtones became clearly resolved. For many of these measurements, the laser system had to maintain a 0.35% RSD over the course of several days. The 9 ps 2D ES often used to guide the reader in the main text, for instance, was taken at hour 30 of a 38-hour scan. These challenges illustrate the success of the NV center measurements and also caution against overinterpretation of 2D ES data measured without sufficient instrumentation advancement. A truncated list of instrument parameters is listed in **Supplementary Table 1** highlighting the three datasets presented in the main paper.

Supplementary Table 1. Table of key parameters for the three measurements presented in the main text. The final NV center measurement was conducted on 09/14/19 with a two-stage NOPA and the machine learning pulse compression, with follow-up measurements contributing to some of the presented spectra. The 05/04/18 spectra were acquired immediately following the conversion of the previous compressor section of the ultrafast spectroscopy setup into a prism-based pulse shaper, enabling more throughput and better chirp compensation.

Date	Power		Pulse Duration (fs)	Bandwidth* (nm)	2D ES Parameters		KCQ**
**07/06/17**	Ave:	52 µW	9.9	514-787	τ_1:	0 to 60 fs in 1 fs steps	200
	Peak:	42 nJ			τ_2:	0 to 1000 fs in 1 fs steps	
**05/04/18**	Ave:	50 µW	8.6	510-720	τ_1:	0 to 75 fs in 1 fs steps	300
	Peak:	40 nJ			τ_2:	0 to 60 ps in 200 fs steps	
**09/14/19**	Ave:	111 µW	6.5	515-770	τ_1:	0 to 90 fs in 1 fs steps	400
	Peak:	88 nJ			τ_2:	0 to 12 ps in 30 fs steps	

*The spectral form of the NOPA output was approximately a top hat interrupted by a peak around 520 nm.
**Kinetic-cycle quads: the number of averages per 2D spectrum.

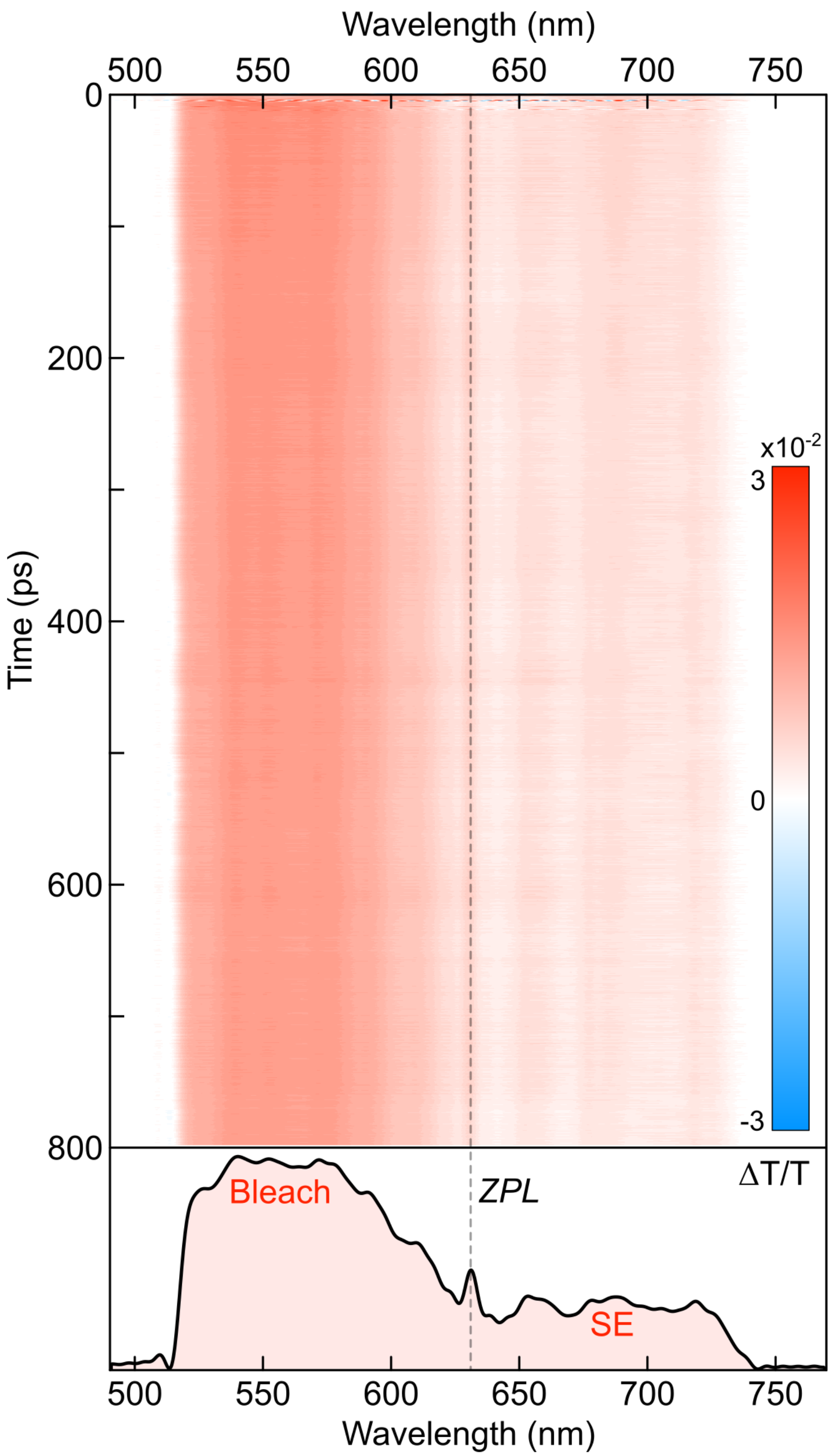

Supplementary Figure 3. Transient absorption (TA) spectrum of the NV center from 0 to 800 ps. The long-timescale TA is unmodulated by any vibronic features, instead showing the long-lived decay of the NV center excited state.

Over the course of working with the NV center material system, it was necessary to occasionally clean the surface of the diamond structure. Degradation of the sample occurs from oxidation of the surface carbon bonds to either a ketone or carboxylic acid derivative, which causes pronounced scatter and reduced signal during ultrafast measurements [5]. As expected from a photoactive sample, the degradation accumulates faster after multiple measurements worth of photoexcitation. The data presented for the NV center stems primarily from four, multi-day measurement trials over the course of three years. For the latter two experiments, which yielded the bulk of the 2D ES, and all of the 3D phase map data, the NV center was cleaned prior to being measured.

The cleaning process was adapted from [6] and involves a straightforward "piranha wash" of the entire NV center sample for a period of 12 hours. The piranha wash is composed of 3 parts concentrated sulfuric acid and 1 part hydrogen peroxide, which hydrolyzes the oxidized C-O bond of the degraded diamond sample and redeposits the carbon onto the surface as either a dangling C-H bond or, more commonly, a C=C double bond. We found that a 12-hour wash in a small vial of gently stirred, boiling, piranha was sufficient to significantly reduce the scatter during the ultrafast

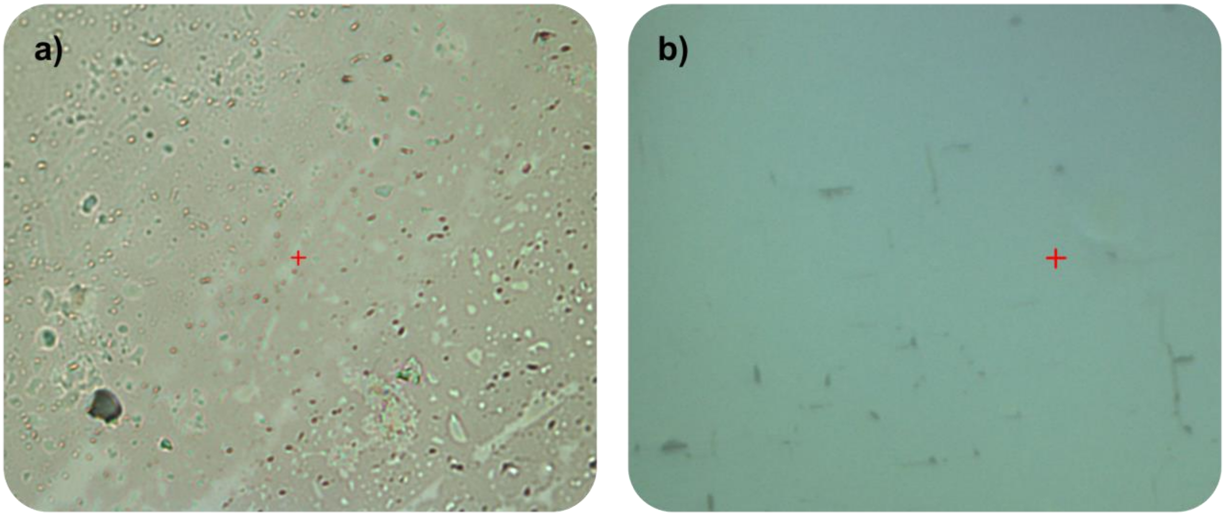

Supplementary Figure 4. Microscope photo of the NV center investigated in this report before (a) and after (b) a 12-hour cleaning in piranha solution. Data taken on a Thermo-DXR2 Raman Microscope with a backlit 100× magnification.

spectroscopy measurements. The washing procedure was conducted in a chemical fume hood, with appropriate neutralization agents located nearby for safety and disposal purposes. As seen in **Supplementary Figure 4**, the reduction of defects on the surface after washing with piranha is observable even with a conventional microscope. The absorption and photoluminescence spectra of the NV center sample were confirmed to be the same before and after the piranha wash, indicating no alteration of the sample's spectroscopic properties.

Supplementary Discussion

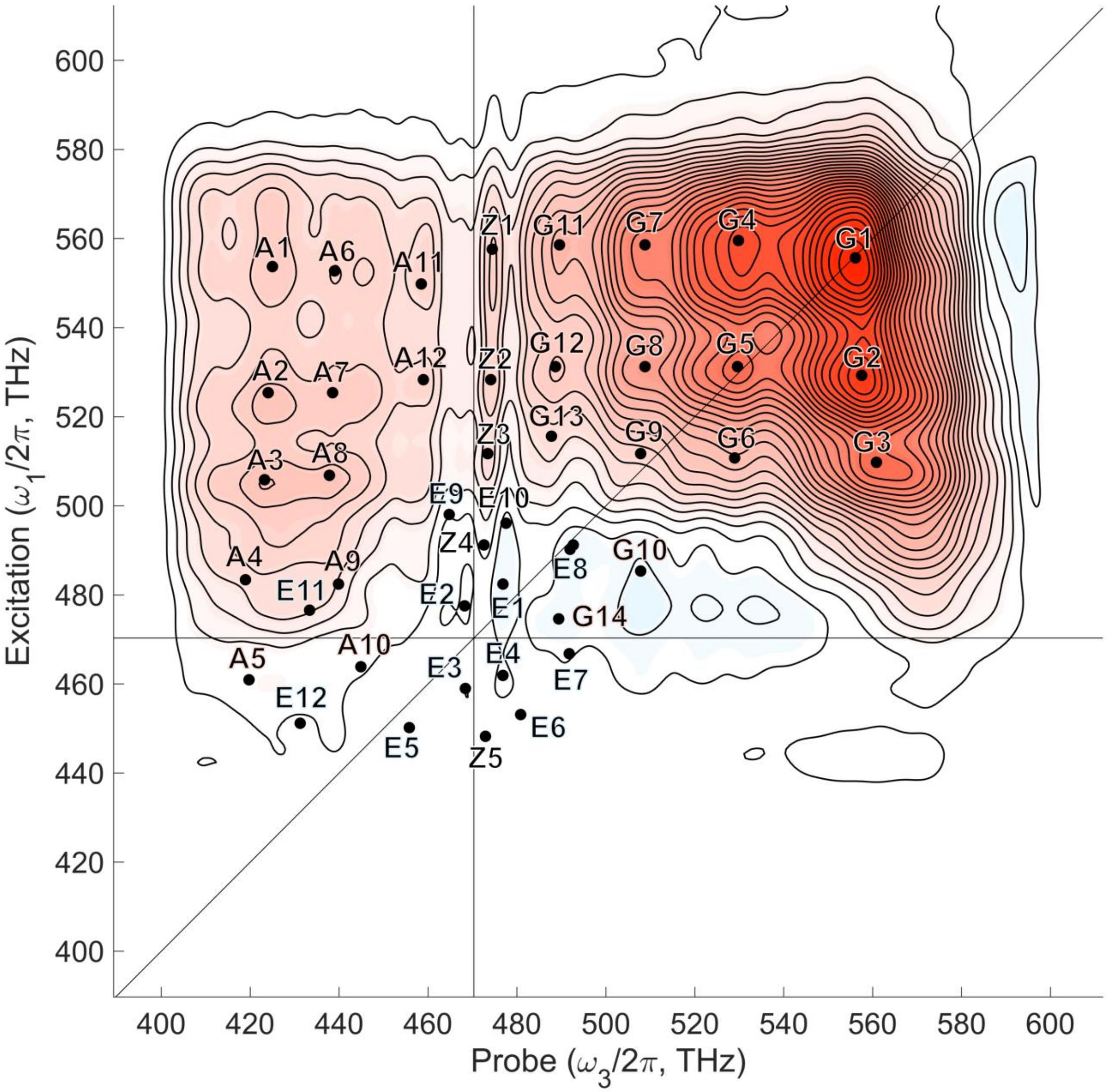

Supplementary Figure 5. Annotated 2D ES of the NV center at 10 ps. The labeled dots correspond to peak locations of interest from 0-12 ps, including the ESA features centered around the ZPL. These peak locations also served as the locations for the temporal analysis summarized in Figure 4 in the main text.

The vibrational analysis of the TA and 2D ES data revealed no contributions to the coherent motion of the NV center by the quasi-local vibrational modes (qLVM) predicted by theory [7]. Instead, the vibrational modulation of the NV center is primarily affected by the LO phonon of the diamond lattice surrounding the defect, and this modulation is particularly pronounced around the Jahn–Teller distortion of the zero-phonon line (ZPL). The predicted qLVMs, however, do appear as 2D ES *vibronic* peaks within the GSB, SE, and ESA regions. By observing a 2D ES at a waiting time corresponding to a peak of the LO oscillation, the excitation and emission energies of these vibronic peaks can be measured and arranged to give a picture of the vibronic energy levels of the NV center. As seen in **Supplementary Figure 5**, we chose a spectrum around 10 ps and identified numerous peaks labeled with **G** for ground-state bleach, **A** for stimulated emission, **E** for excited-state absorption, and **Z** for the zero-phonon line.

Supplementary Table 2. The table of vibronic energy levels assembled from the energy difference between discrete peaks in the 2D spectrum at 10 ps. The analysis is separated into the ground-state bleach (GSB), stimulated emission (SE), and zero-phonon line (ZPL) spectral regions, with blue shading representing emission levels and orange shading representing excitation levels. Energies are given in THz and meV, with THz appearing closer to the peak difference label.

						ZPL Region							
			Average	Std. Dev.	Mode	meV	Z2-Z1	29.3	121.2				
E10-E8	15.14	62.61	15.03	0.11	11.78	48.7	Z3-Z2	16.6	68.7				
E4-E7	14.92	61.69	62.15	0.46	2.94	12.18	Z4-Z3	20.5	84.8				
							Z4-ZPL	17.2	71.2				
E9-E10		0.00	Average	Std. Dev.						Average	Std. Dev.		
E1-E2	8.62	35.64	8.53	0.08			E3-E2	18.6	76.7	19.53	0.98		
E3-E4	8.45	34.94	35.29	0.35			E4-E1	20.5	84.8	80.8	4.0		
										Average	Std. Dev.		
E9-ZPL		0.00	Average	Std. Dev.			E5-E3	8.8	36.3	8.8	0.0		
E2-ZPL	5.77	23.86	5.69	0.08			E6-E4	8.8	36.3	36.3	0.0		
E3-ZPL	5.60	23.17	23.5	0.3						Average	Std. Dev.		
							E5-ZPL	23.8	98.4	22.33	1.46		
E3-E5	-12.61	-52.16					E6-ZPL	20.9	86.3	92.4	6.1		
										Average	Std. Dev.		
E6-E4	3.99	16.51					E10-E1	20.51	84.8	22.46	1.95		
			Average	Std. Dev.			E9-E2	24.41	101.0	92.9	8.1		
Z5-ZPL	1.08	4.46	1.25	0.17									
Z4-ZPL	1.42	5.85	5.2	0.7			E12-E11	25.4	105				
						GSB Region							
G1-G4	-26.32	-108.85	Average	Std. Dev.	Mode	meV	G2-G1	26.4	109	Average	Std. Dev.	Mode	meV
G2-G5	-27.94	-115.56	-28.72	2.34	-53.05	-219.4	G5-G4	28.3	117	27.3	0.7	69.0	285
G3-G6	-31.90	-131.91	-118.8	9.7	-13.26	-54.85	G8-G7	27.3	113	113	3	69.0	285.3
							G11-G12	27.3	113				
G4-G7	-21.02	-86.94	Average	Std. Dev.	Mode	meV							
G5-G8	-20.81	-86.05	-20.99	0.14	-24.33	-100.6	G3-G2	19.5	80.8	Average	Std. Dev.	Mode	meV
G6-G9	-21.15	-87.45	-86.82	0.58	-8.11	-33.54	G6-G5	20.5	84.8	18.8	1.9	41.6	172
							G9-G8	19.5	80.8	77.7	7.8	41.6	172.2
G7-G11	-19.23	-79.55	Average	Std. Dev.	Mode	meV	G13-G12	15.6	64.6				
G8-G12	-20.14	-83.28	-18.83	1.74	-3.34	-13.8							
G9-G13	-20.06	-82.96	-77.87	7.18	-1.67	-6.90	G10-G3	24.4	101.0	Average	Std. Dev.	Mode	meV
G10-G14	-15.88	-65.69					G10-G6	25.4	105.0	11.5	24.1	22.8	94
							G10-G9	26.4	109.0	47.5	99.7	22.8	94.4
G11-ZPL	15.57	64.39	Average	Std. Dev.			G10-G13	-30.3	-125.2				
G12-ZPL	14.67	60.67	15.49	1.56									
G13-ZPL	13.77	56.95	64.06	6.43			G10-ZPL	11.4	47.0				
G14-ZPL	17.95	74.23											
SE Region													
A1-A6	13.98	58	Average	Std. Dev.	Mode	meV	A1-A2	28.3	117	Average	Std. Dev.	Mode	meV
A2-A7	14.50	60	14.35	0.3	69.7	288	A6-A7	27.3	113.1	25.7	3.0	69.7	288
A3-A8	14.59	60	59.36	1	69.7	288.1	A11-A12	21.5	88.9	106.4	12.5	69.7	288.1
			Average	Std. Dev.	Mode	meV				Average	Std. Dev.	Mode	meV
A11-A6	19.50	81	19.96	0.5	43.9	182	A2-A3	19.53	80.8	20.5	1.0	43.9	182
A12-A7	20.42	84	82.54	2	43.9	181.7	A7-A8	21.48	88.8	84.8	4	43.9	181.7
			Average	Std. Dev.	Mode	meV				Average	Std. Dev.	Mode	meV
A11-ZPL	15.52	64	15.28	0.2	23.4	97	A3-A4	22.46	93	23.4	1.0	23.4	97
A12-ZPL	15.04	62	63.21	1	23.4	96.9	A9-A8	24.41	101	97	4	23.4	96.9

Measuring the difference between each peak in the excitation and emission dimensions results in **Supplementary Table 2**. The difference between any two peaks represents the energy difference between the vibronic energy levels of the NV center. For instance, the difference between the emission locations of G7 and G11 is 19.23 THz (79.55 meV) and corresponds to the 2nd vibronic level of an E-symmetric vibrational mode on the excited state of the NV center. In

molecular samples, this analysis would be confounded somewhat by the lack of a discrete difference between the lowest-energy excited state and lowest-energy ground state. The ZPL of the NV center, however, provides a true zero point from which to build out a ladder of vibronic levels. Averaging the difference between each sequential pair of 2D ES peaks returns the fundamental vibronic level, usually to within a 3 THz standard deviation. It is these averaged vibronic modes that we compare to the theoretical predictions for the qLVMs.

A comparison of the vibronic modes obtained by the vibronic analysis described above and the qLVMs predicted by theory is presented in **Supplementary Table 3**. The *ab initio* calculations conducted by Abtew, *et. al.* [7] involved two scenarios with strong ($G \neq 0$) and weak ($G=0$) electronic coupling to the Jahn–Teller vibrational mode. The energy spacings of the peaks in the 2D ES returned an average value of 36.3 meV (8.8 THz) in the ZPL region, corresponding well to the predicted energy of the Jahn–Teller vibrational mode with strong electronic coupling.

In fact, the vibronic analysis returned energy levels within 1 THz (4.1 meV) of all the theoretically predicted qLVMs across both the A1 and E symmetry groups. We also confirmed three of the vibrational modes obtained by Ulbricht, *et. al.* [8] from a power spectrum of a narrowband pump-probe experiment that measured the Jahn-Teller induced electronic depolarization of the NV center. It is notable that none of the power spectra obtained in our analysis of the TA, 2D, and 3D ES data returned peaks with energies corresponding to qLVM energies predicted by theory. This discrepancy between the vibronic analysis and the power spectra gives rise to the assertion in the main text that the LO phonon modulates the Jahn–Teller vibrational mode and geometric distortion, while the qLVMs represent a vibronic energy level ladder resting on top of this modulation.

Supplementary Table 3. A comparison table of the vibrational modes found in this study using the vibronic analysis described in this section and the power spectra of the TA and 3D ES data described in the main text. Grey highlighting indicates red-shifts, and other splittings, while blue shading represents vibrational modes unique to this study. Comparisons are made to the power spectra obtained from Ulbricht, *et. al.* [8] and Abtew *et. al* [7] in the strong (G≠0) and weak (G=0) coupling regimes.

	Comparison Table						
	**ID**	*Energy (meV)*	*Frequency (THz)*	*Ulbricht (2016)*	*Abtew, G ≠ 0 (2011)*	*Abtew, G = 0 (2011)*	*Symmetry*
Vibronic Analysis	v'	39.0	9.4		36.3	39.3	E
	v_1	23.6	5.7				
	v_2	28.8	7.0				
	v_3	36.3	8.8		36.7	39.3	E
	v_4	47.5	11.5	47			
	v_5	57.2	13.8				
	v_6	66.2	16.0	69	67.1		A1
	v_7	75.0	18.1		78.1		E
	v_8	85.3	20.6	90		88.5	E
	v''	95.9	23.2				
Power Spectra	v_9	9.03	2.18				
	v_{10}	11.5	2.8				
	v_{11}	15.2	3.7				
	v_{12}	17.9	4.3				
	v_{13}	19.0	4.6				
	v_{14}	133	32	130	129.4		A1
	v_{LO}	166	40		162.9		A2
	$2v_{LO}$	331	80				
	$3v_{LO}$	488	118				
	$4v_{LO}$	649	157				
	$2v$-$2v$	9	2				
	v_{AN}	5	1				

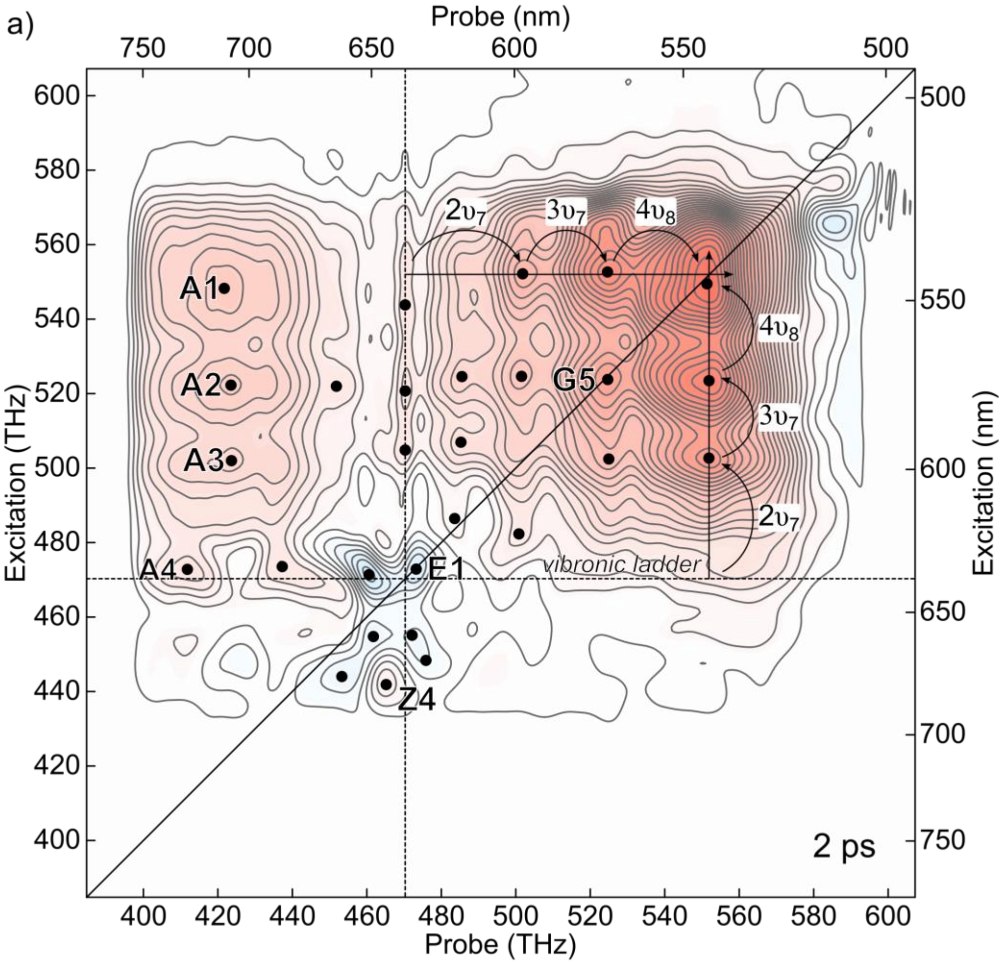

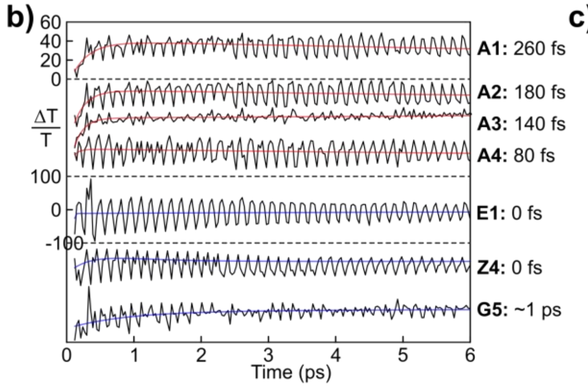

c)

ID	Peak Separation THz (meV)	Ulbricht	Abtew
υ'	9.43 (39.0)		9.50 (39.3)
$υ_1$	5.70 (23.6)		
$υ_2$	6.97 (28.8)		
$υ_3$	8.79 (36.3)		8.87 (36.7)
$υ_4$	11.5 (47.5)	11 (47)	
$υ_5$	13.8 (57.2)		
$υ_6$	16.0 (66.2)	17 (69)	16.2 (67.1)
$υ_7$	18.1 (75.0)		18.9 (78.1)
$υ_8$	20.6 (85.3)	22 (90)	21.4 (88.5)
υ''	23.1 (95.9)		

Supplementary Figure 6. Excited-state energy relaxation of the NV center. (a) Peak locations overlaid on a 2D electronic spectrum with a waiting time of 2 ps were distributed across the bleach, SE, and ESA regions. (b) Rates obtained from around the ZPL region and fitted to biexponential decays confirm ultrafast relaxation decay through the JT conical intersection. (c) Expanded table of vibronic peaks found using the vibronic analysis.

The following figures include additional 2D ES that may be of interest. Some of these spectra were taken prior to implementation of the two-stage NOPA and machine-learning enabled pulse compressor. The clear vibronic peaks presented in the main text are therefore suppressed, although the observed dynamics of the NV center remained consistent over the course of all measurements.

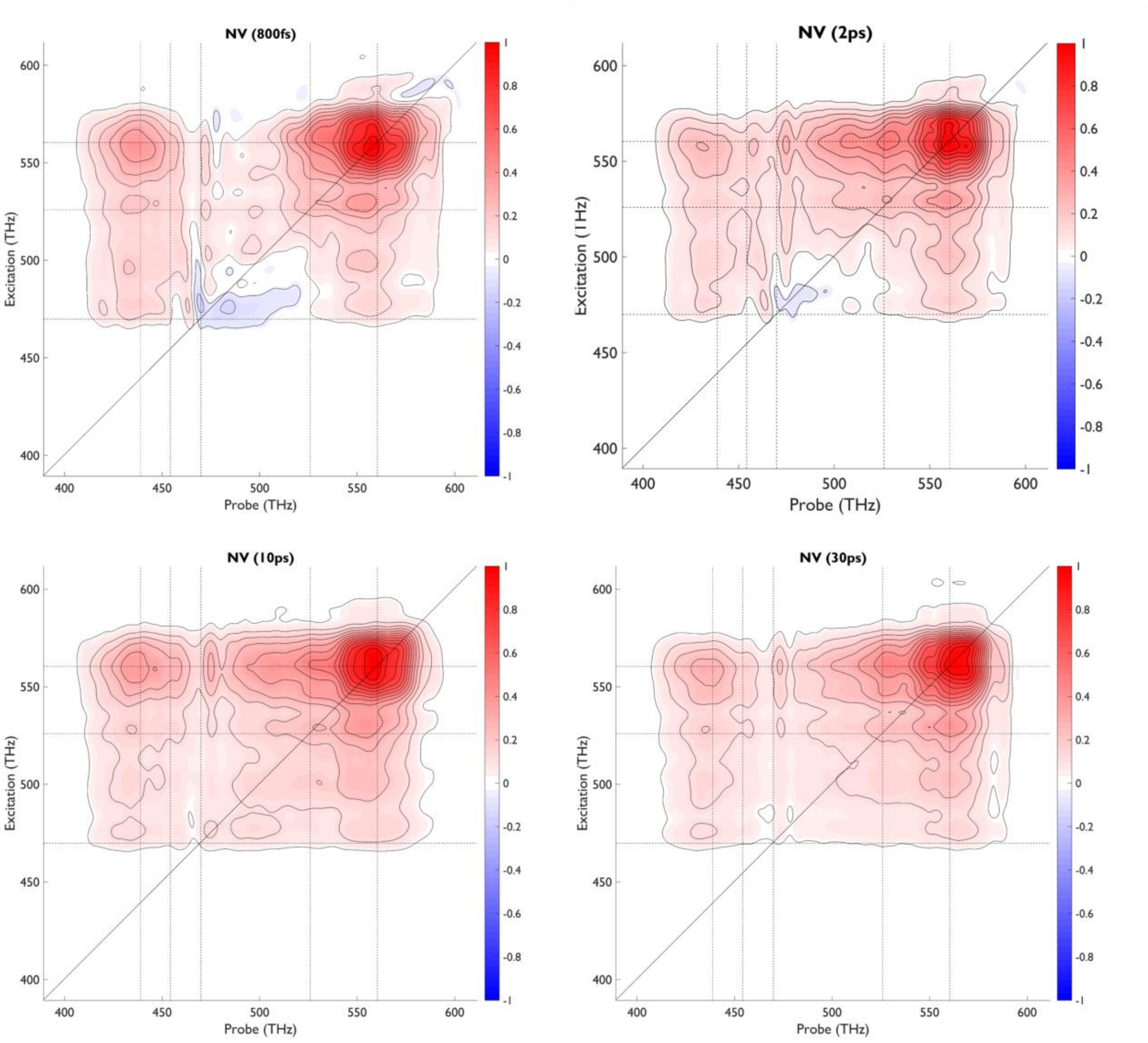

Supplementary Figure 7. A collection of 2D ES showing the gradual decay of the ESA features centered on the ZPL over the course of 30 ps.

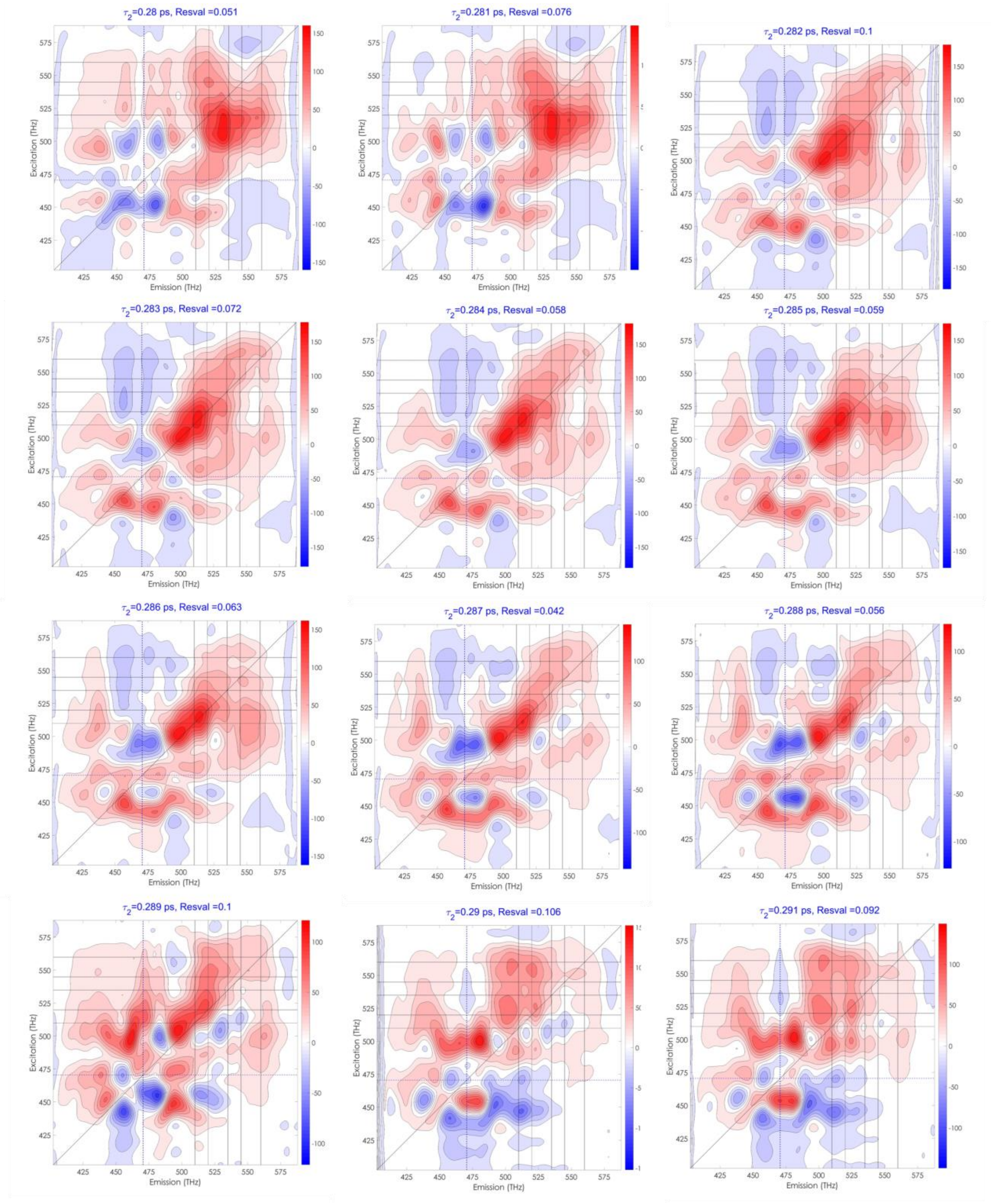

Supplementary Figure 8. A selection of 2D ES from 280 fs to 291 fs showing the LO phonon modulation of the ZPL region and delayed stimulated emission. The LO phonon has a period of 25 fs, half of which is represented here. The "resval" is the measure of the 2D spectrum's fit to its corresponding TA trace, with values under 0.1 considered to be "well-phased" for the NV center.

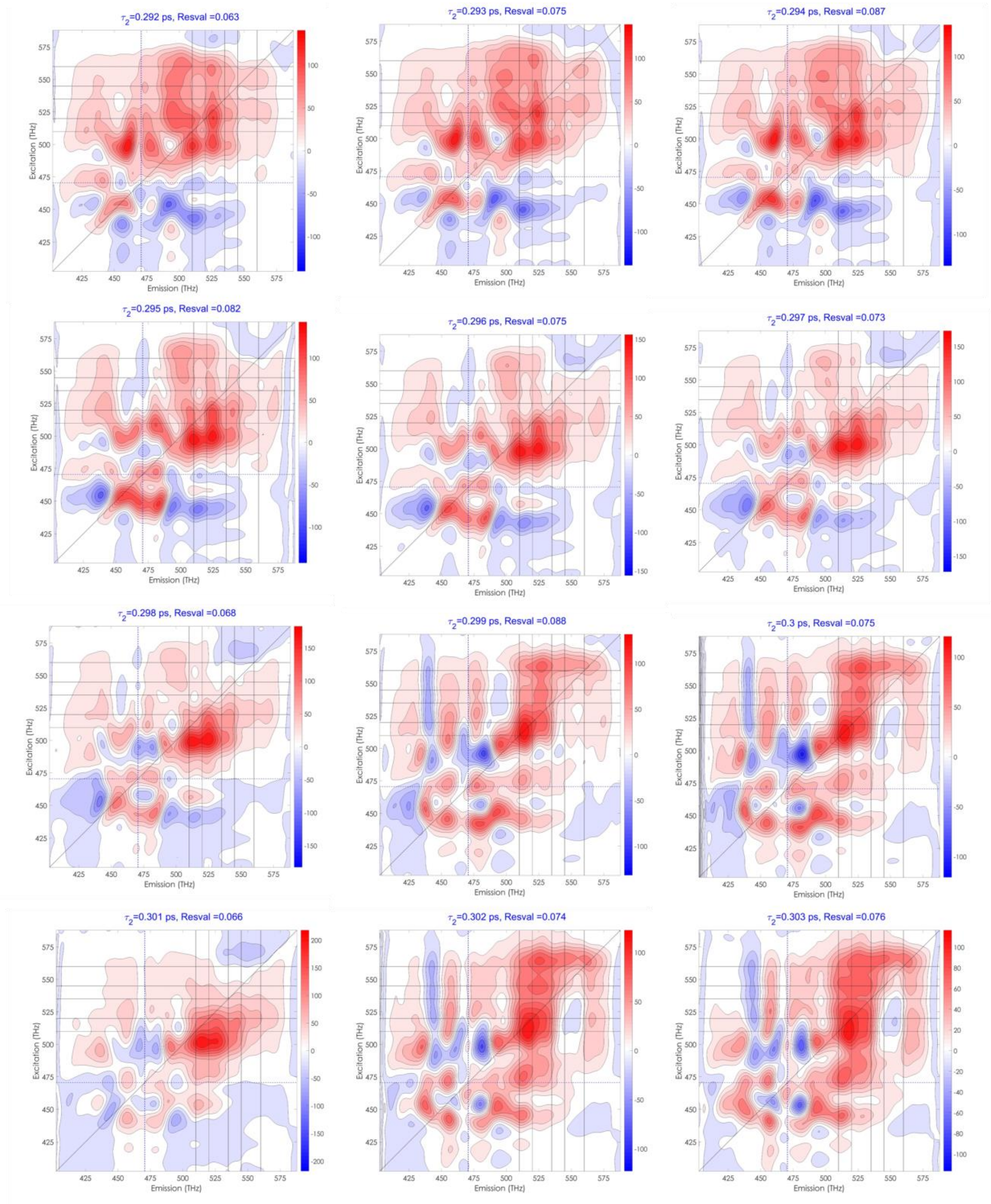

Supplementary Figure 9. The second half of the LO phonon modulation from 292 fs to 303 fs. The "resval" is the measure of the 2D spectrum's fit to its corresponding TA trace, with values under 0.1 considered to be "well-phased" for the NV center.

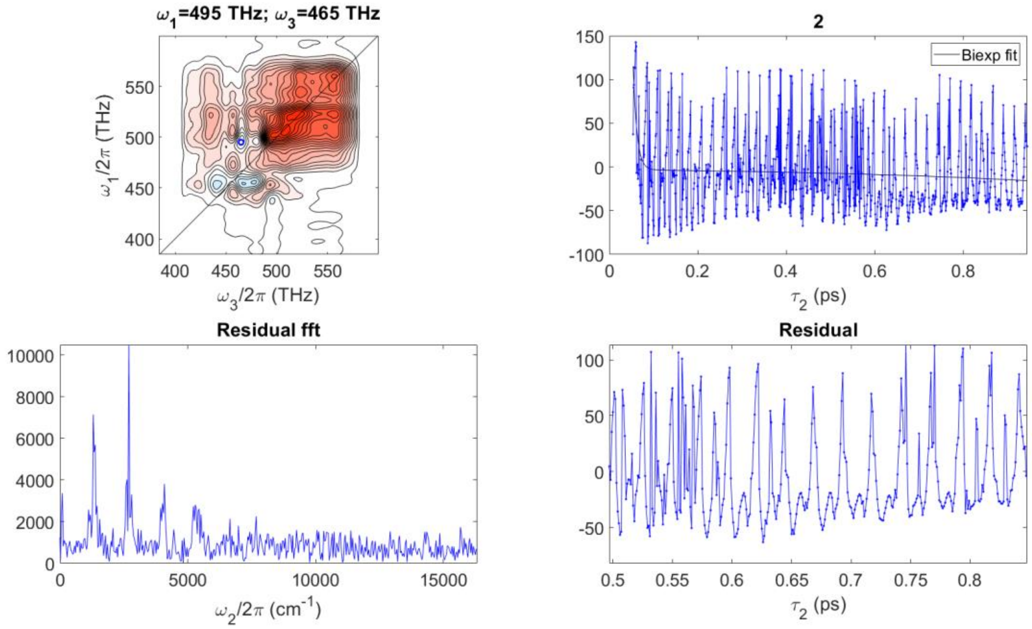

Supplementary Figure 10. An example of the 3D ES data-analysis process used to generate the phase maps and power spectra presented in the main text. Excitation and Emission points on a 3D dataset (top left) are fit to a biexponential decay function (top right). The residual (bottom right) is then Fourier transformed to return a power spectrum (bottom left) corresponding to the coherent motion of that coupled electronic transition. In this specific example, the excited-state absorption (ESA) feature near the ZPL was analyzed to reveal LO overtone vibrations in the 3D power spectrum.

Supplementary References

[1] Doherty, M. W.; Manson, N. B.; Delaney, P.; Jelezko, F.; Wrachtrup, J.; Hollenberg, L. C. L. “The Nitrogen-Vacancy Colour Centre in Diamond”. *Physics Reports*. **2013**, (528), 1–45.

[2] Brazard, J. Bizimana, L. A.; Turner, D. T., Accurate Convergence of Transient-Absorption Spectra Using Pulsed Lasers. *Rev. Sci. Instrumen.* **2015**, *86* (5), 053106.

[3] Bizimana, L. A.; Brazard, J.; Carbery, W. P.; Gellen, T.; Turner, D. B. “Resolving Molecular Vibronic Structure Using High-Sensitivity Two-Dimensional Electronic Spectroscopy”. *J. Chem. Phys.* **2015**, *143* (16), 164203.

[4] Farfan, C. A.; Epstein, J.; Turner, D. B. “Femtosecond Pulse Compression using a Neural-Network Algorithm”. Optics Letters. **2018**, (43), 5166–5169.

[5] Sangtawesin, S.; Dwyer, B. L.; Srinivasan, S.; Allred, J. J.; Rodgers, L. V. H.; De Greve, K.; Stacey, A.; Dontschuk, N.; O’Donnel, K. M.; Hu, D.; Evans, D. A.; Jaye, C.; Fischer, D. A.; Markham, M. L.; Twitchen, D. J.; Park, H.; Lukin, M. D.; de Leon, N. P. “Origins of Diamond Surface Noise Probed by Correlating Single-Spin Measurements with Surface Spectroscopy”. *Phys. Rev. X*. **2019**, (9), 031052.

[6] Cooper, A.; Sun, W. K. C.; Jaskula, J.-C.; Cappellaro, P.; “Identification and Control of Electron-Nuclear Spin Defects in Diamond”. *Phys. Rev. Lett*. **2020**, (124), 083602.

[7] Abtew, T. A.; Sun, Y. Y.; Shih, B.-C.; Dev, P.; Zhang, S. B.; Zhang, P. “Dynamic Jahn-Teller Effect in the NV− Center in Diamond”. *Phys. Rev. Lett*. **2011**, (107), 146403.

[8] Ulbricht, R.; Dong, S.; Chang, I.-Y.; Mariserla, B. M. K.; Dani, K. M.; Hyeon-Deuk, K.; Loh, Z.-H. “Jahn-Teller-Induced Femtosecond Electronic Depolarization Dynamics of the Nitrogen-Vacancy Defect in Diamond”. *Nature Communications*. **2016**, (7), 13510.
